# Phase Behavior of Thermoresponsive Colloids Drives Re‐Entrant Plasmon Coupling

**DOI:** 10.1002/nap2.70322

**Published:** 2026-11-13

**Authors:** Angela Capocefalo, Francesco Brasili, Javier Pérez, Edouard Chauveau, Stefano Casciardi, Andrea Militello, Francesco Sciortino, Emanuela Zaccarelli, Federico Bordi, Domenico Truzzolillo, Simona Sennato

**Affiliations:** ^1^ Institute for Complex Systems National Research Council Roma Italy; ^2^ Physics Department Sapienza University of Rome Roma Italy; ^3^ Synchrotron SOLEIL L'Orme des Merisiers Saint‐Aubin France; ^4^ Laboratoire Charles Coulomb CNRS–Université de Montpellier Montpellier France; ^5^ Dipartimento di Medicina, Epidemiologia, igiene del lavoro e ambientale Istituto Nazionale per l’Assicurazione contro gli Infortuni sul Lavoro Roma Italy

**Keywords:** colloidal stability, plasmon coupling, re‐entrant condensation, soft matter photonics, stimuli‐responsive nanomaterials, thermoresponsive microgels

## Abstract

Plasmonic nanoparticles (NPs) integrated within thermoresponsive polymeric microgels provide a versatile platform for the realization of stimuli‐responsive optical materials, where the microgel volume phase transition enables dynamic control of plasmon coupling. This study uncovers a counter‐intuitive re‐entrant behavior with increasing NP loading in which plasmon coupling initially strengthens and subsequently weakens beyond a critical NP‐to‐microgel number ratio. By combining light and X‐ray scattering techniques with optical spectroscopy and electrophoretic mobility measurements, it is demonstrated that plasmon coupling is governed not only by the interparticle distance between NPs confined within individual microgels but also by the colloidal stability of the hybrid complexes. At intermediate NP loadings, surface charge inhomogeneities induced by NP adsorption promote the aggregation of microgel–NP complexes, resulting in enhanced plasmon coupling. In contrast, when the complexes remain colloidally stable, coupling is dictated solely by NP organization within the corona of individual microgels. A quantitative relationship between plasmon coupling and interparticle distance reveals two distinct coupling regimes. This behavior is rationalized through a phase diagram linking colloidal stability to optical response. These findings identify colloidal stability as a key parameter for designing soft plasmonic systems with programmable optical properties.

## Graphical abstract

Plasmonic nanoparticles assembled onto thermoresponsive microgels display a counter‐intuitive re‐entrant plasmon coupling as a function of nanoparticle loading. A phase diagram linking the colloidal stability of the microgel–nanoparticle complexes to their optical response reveals two distinct coupling regimes, governed by charge‐patch‐driven aggregation. These findings identify colloidal stability as a key design parameter for soft plasmonic materials with programmable optical properties.

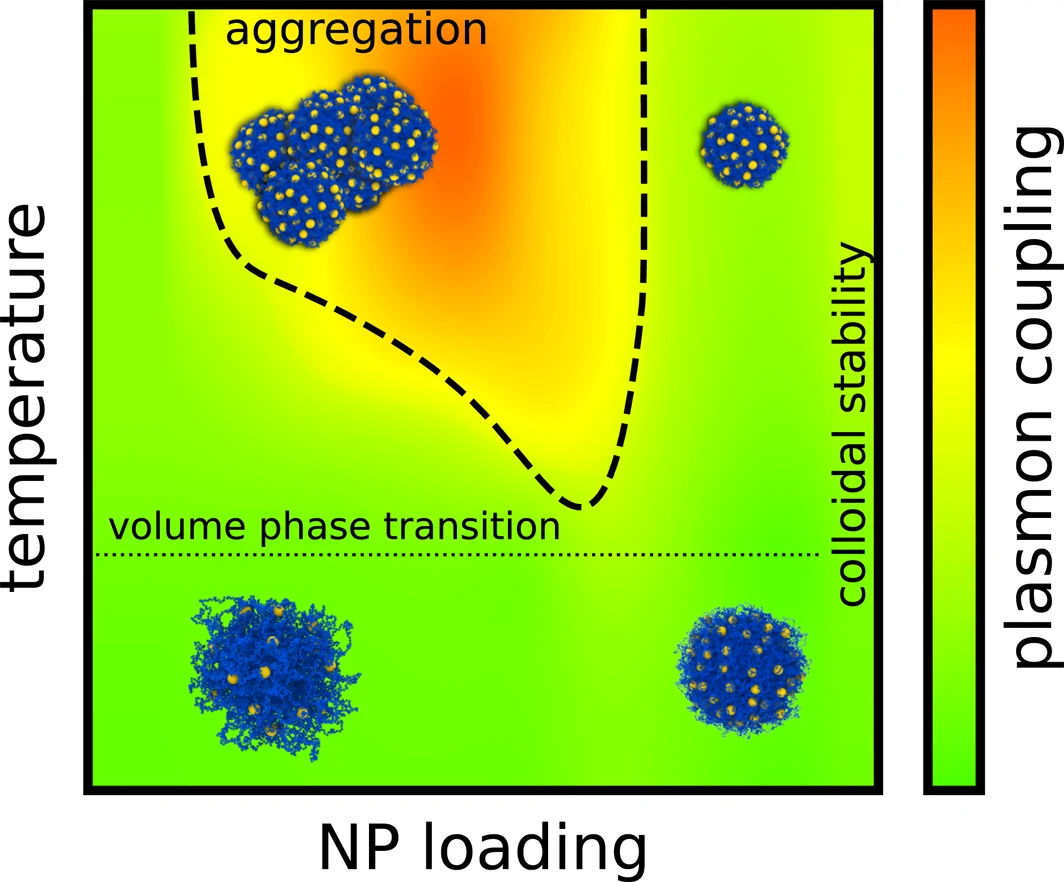

## 1 Introduction

The integration of plasmonic nanoparticles (NPs) with soft polymeric colloids, such as microgels, has emerged as a convenient strategy for designing smart photonic materials with tunable optical properties [1, 2, 3]. The unique and programmable features of these systems arise from the interplay between the dual colloidal‐polymeric nature of microgels [4, 5], which enables precise control over softness and responsiveness to external stimuli, and the distinctive optical properties of the NPs [6, 7, 8]. In particular, noble metal NPs exhibit resonant interaction with visible light, at the so‐called localized surface plasmon resonance (LSPR), whose spectral features are highly sensitive to interparticle spacing and organization through plasmon coupling effects, which occur when interparticle gaps fall within a few nanometers [9].

Thermoresponsive microgels offer a versatile platform to reversibly modulate NP organization via their volume phase transition (VPT), that is, the collapse of the polymer network upon increasing temperature. The VPT has been widely exploited as a mechanism to bring NPs in close proximity and thereby control plasmon coupling [10, 11, 12, 13, 14, 15], establishing microgel–NP complexes as attractive platforms for sensing, photothermal therapy, or nanocatalysis [16, 17, 18, 19, 20]. These features place microgel–NP complexes within the broader context of soft matter photonics, which exploits the intrinsic flexibility of soft materials to control light–matter interactions [21, 22]. Such hybrid systems provide novel routes for manipulating light at sub‐diffraction length scales, highlighting the importance of understanding how their properties can be optimized to achieve the desired optical response.

The simplest and one of the most commonly used strategies to incorporate NPs within microgels relies on electrostatic attraction between the NPs and oppositely charged groups within the polymer network [18, 23, 24, 25]. Once adsorbed, stronger short‐range interaction between the metal surface and the polymer could help anchor and stabilize the NPs [12]. We recently investigated in detail these electrostatic interactions between thermoresponsive poly(N‐isopropylacrylamide) (pNIPAM) microgels and gold NPs, addressing both the impact of NP adsorption on the structure of the polymer network [26], as well as the spatial organization of NPs within the spherical corona of the microgel and their rearrangement driven by the microgel VPT [15]. We also established a quantitative relationship between plasmon coupling and thermally tunable NP spacing. However, the nonuniform surface charge distribution, resulting from NP adsorption, can lead to unexpected aggregation phenomena, including finite‐size clustering and overcharging, that is the reversal of the sign of the net charge of the complexes compared to bare microgels, which results in a re‐entrant condensation [27]. This nontrivial phenomenology has been previously observed separately in both microgel‐based systems, where thermoresponsive microgels were decorated by smaller species [28, 29] and plasmonic NPs decorated by globular proteins [30, 31]. Instead, the role of colloidal stability in determining the optical properties of microgel–NP complexes has remained largely unexplored. Previous studies have primarily focused on tuning plasmon coupling by varying the microgel crosslinker density or the NP surface coverage [11, 12], while largely overlooking the colloidal behavior of the resulting hybrid complexes.

In this work, we tackle this problem by systematically analyzing the optical properties of microgel–NP complexes across the VPT while varying the NP‐to‐microgel number ratio n. We reveal a counter‐intuitive modulation of plasmon coupling, which initially strengthens with increasing n and then weakens again beyond a critical threshold. To rationalize these findings, we report a small angle X‐ray scattering (SAXS) analysis of the NP–NP correlation to establish a direct, quantitative connection between NP gathering across VPT and the resulting optical response of the system in terms of plasmon coupling. We find a dual behavior depending on n, that we finally explain within the framework of finite‐size clustering and re‐entrant condensation of charge‐patched colloids, through hydrodynamic radius, electrophoretic mobility, and electron microscopy measurements.

We unveil a re‐entrant plasmon coupling in the microgel–NPs system that is governed primarily by complexes aggregation, in addition to the number and relative positioning of NPs adsorbed within the microgel corona. By identifying the key parameters controlling plasmon coupling in dependence of the colloidal properties of the system, this work establishes a rational framework for tuning the optical properties of these hybrid plasmonic systems, thus paving the way toward the design of responsive plasmonic materials with programmable optical properties for sensing, photonic, and nanomedicine‐related applications.

## 2 Results and Discussion

### 2.1 Thermal Modulation of the Optical Response

We first investigate the thermal modulation of the optical properties of microgel–NP complexes with different NP‐to‐microgel number ratios n by extinction spectroscopy. The complexes are obtained by an electrostatic adsorption of anionic gold NPs, with radius a=9.3 nm and LSPR peak at $\lambda_{\text{NP}}=525$ nm, to cationic microgels with VPT at $T_{\text{VPT}}=32$°C. Control spectra of stock NPs and pristine microgels dispersions, reported in Supporting Information S1: Figure S1, demonstrate that the contribution to the extinction due to scattering from microgels [32] is negligible compared to the LSPR of NPs, even in the collapsed state. The spectra for selected values of n are shown in in Figure 1, and additional datasets are reported in Supporting Information S1: Figure S2. At 25°C, below $T_{\text{VPT}}$, no signs of plasmon coupling can be detected, as all LSPR peaks exhibit the typical spectral shape of isolated, noninteracting NPs. As temperature increases, the spectra evolve, exhibiting both a redshift of the LSPR peak and an increase in optical extinction at longer wavelengths (λ≥570 nm). These spectral changes are characteristic of plasmon coupling [6]; therefore, one would be tempted to attribute them to the progressive gathering of adsorbed NPs, driven by the shrinking of microgels across the VPT. Under this interpretation, the coupling would be expected to become progressively stronger with increasing n, as the reduced surface‐to‐surface distance d for higher NP loading (growing with n [15]) would result in stronger plasmon coupling. Instead, the spectral features of plasmon coupling appear to be most evident at intermediate coverage (20≲n≲100) and become less pronounced for the highest n values. We take a deeper look at this unexpected behavior by reporting the LSPR shift $\Delta \lambda_{\text{LSPR}}$ with respect to $\lambda_{\text{NP}}$, and the degree of plasmon coupling $\Delta \left(A_{C}/A_{\mathrm{tot}}\right)$, defined in Equation (1), as a function of n for all the analyzed temperatures in Figure 2a,b, respectively. Both quantities increase with T at fixed n, whereas, at fixed T, they display a nonmonotonic dependence on n. In particular, for temperatures above the VPT, they increase up to n≃50 and then decrease back for higher values of n. Below the VPT, this effect is shifted to larger n and barely noticeable.

**FIGURE 1:**
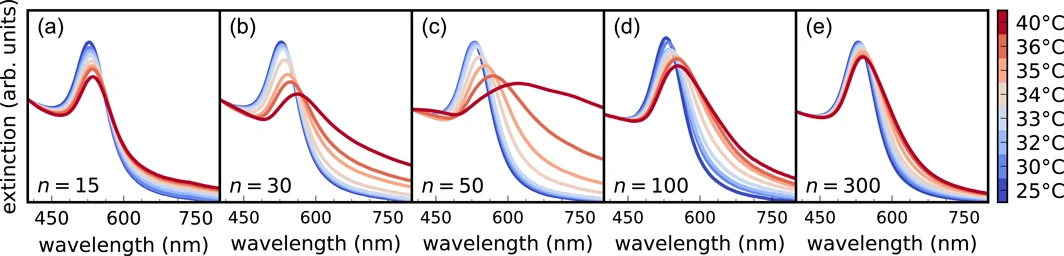
Extinction spectra of microgel–NPs as a function of temperature for selected values of the NP‐to‐microgel number ratio n: 15 (a), 30 (b), 50 (c), 100 (d), and 300 (e).

**FIGURE 2:**
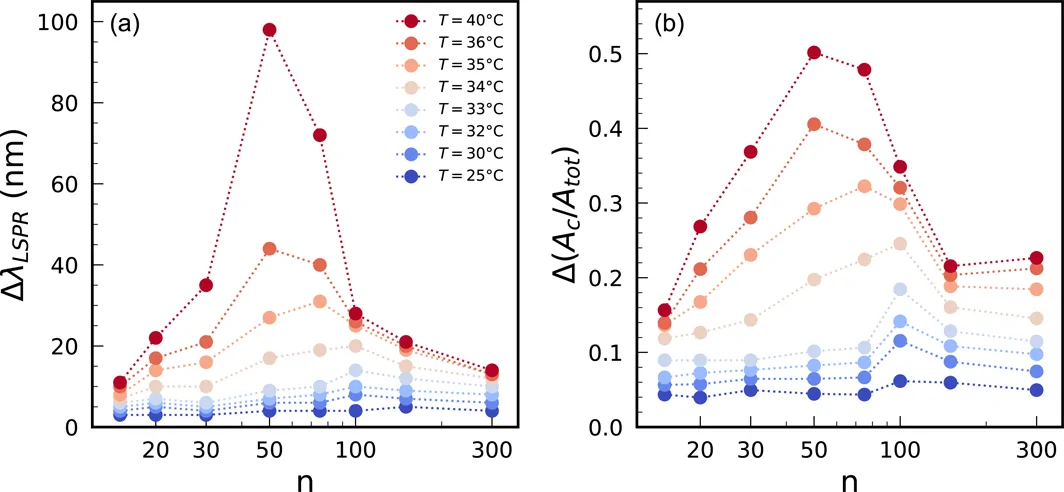
(a) Shift of the LSPR peak $\Delta \lambda_{\text{LSPR}}$ and (b) degree of plasmon coupling $\Delta \left(A_{C}/A_{\mathrm{tot}}\right)$, defined as the spectral weight of the region of coupled plasmon modes λ>570 nm, Equation (1), as a function of n for different T.

These results demonstrate a re‐entrant behavior of the optical properties, which has not been previously observed and thus deserves a dedicated investigation to provide a proper interpretation and identify the underlying mechanisms of plasmon coupling. In this respect, although $\Delta \lambda_{\text{LSPR}}$ is useful for applications such as colorimetric sensing, it has two key limitations, being sensitive to changes in the dielectric environment caused by water expulsion across the VPT, and failing to fully capture the emergence of coupled plasmon modes at longer wavelengths. In contrast, the degree of coupling accounts for the entire spectral modifications and thus better represents the plasmon hybridization that occurs upon the NP approach. In the following section, we therefore combine SAXS measurements with optical analysis to establish a quantitative relationship between plasmon coupling and NP structural arrangement within the microgels, using $\Delta \left(A_{C}/A_{\mathrm{tot}}\right)$ as the key optical parameter.

### 2.2 Linking Optical Properties to NP Structure

To gain knowledge on the microscopic organization of NPs adsorbed to microgels, we study the thermal evolution of their structure factors S(q) for different n. Measurements are performed by SAXS, thus ensuring that the measured intensity originates exclusively from the NPs, thanks to their much higher contrast compared to the polymer [33]. The corresponding S(q) is shown in Figure 3 for the same values of n selected for Figure 1, and additional datasets are reported in Supporting Information S1: Figure S3. The structure factors exhibit several features resulting from the complex structure of the NPs embedded within microgels, whose thermal evolution has a nontrivial interpretation that we provided in a previous work with the aid of numerical simulations [15]. This interpretation involves the formation of a shell of NPs adsorbed in the outer corona of the microgel, which becomes more compact and narrow, driven by the VPT of the microgel. In this context, it is possible to identify two distinct q‐regions, approximately below and above 0.1 ${\text{nm}}^{-1}$, associated with structural correlations at different length scales. Here, we are interested in the surface‐to‐surface distance d between nearest neighbor NPs as a function of temperature; therefore, we focus on the position $q_{p}$ of the first peak in the high‐q range, following the methodology of our previous study. The evolution of $q_{p}$—approximately between 0.1 and 0.3 ${\text{nm}}^{-1}$, depending on n—is highlighted in Figure 3 by light green regions. The average surface‐to‐surface distance between NPs d(n,T), given by Equation (3) for each experimental condition (n,T), is reported in Figure 4a. As expected, the data show that d decreases as temperature increases for all the investigated values of n, reflecting NP gathering driven by the VPT. At fixed T, d also decreases with increasing n, consistent with the larger number of NPs adsorbed to the microgels. However, the data do not appear to correlate with the re‐entrant behavior observed in the optical properties. To explore this aspect, we plot $\Delta \left(A_{C}/A_{\mathrm{tot}}\right)$ as a function of d in Figure 4b. At large d values, the data collapse onto a single master curve. As d decreases, they split into two distinct regimes. Surprisingly, the same interparticle separations correspond to markedly different coupling strengths. In the weaker‐coupling branch, the data for n=15 are in excellent agreement with those for n>100 reported in Brasili et al. [15], whereas all intermediate n data fall on a second branch exhibiting significantly stronger coupling.

**FIGURE 3:**
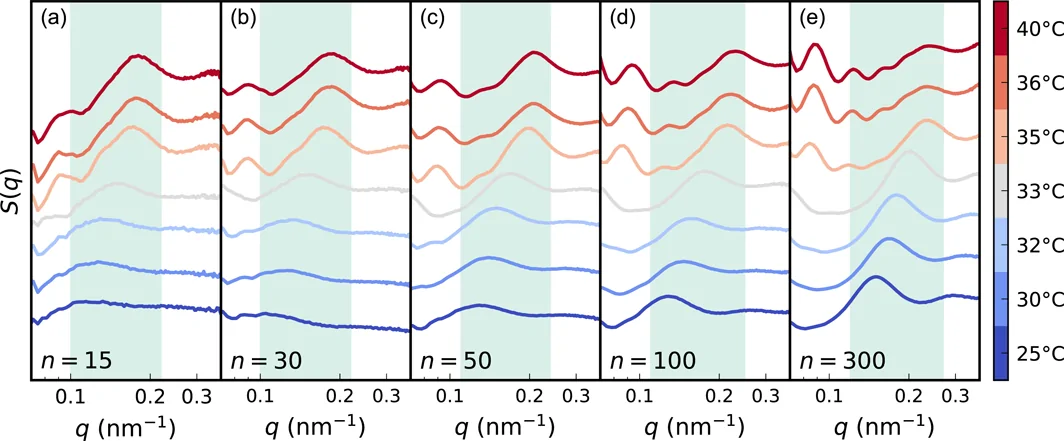
Structure factors of the microgel‐NPs samples as a function of temperature for the same values of n of Figure 1. The light green color highlights the peaks corresponding to the distance between NPs.

**FIGURE 4:**
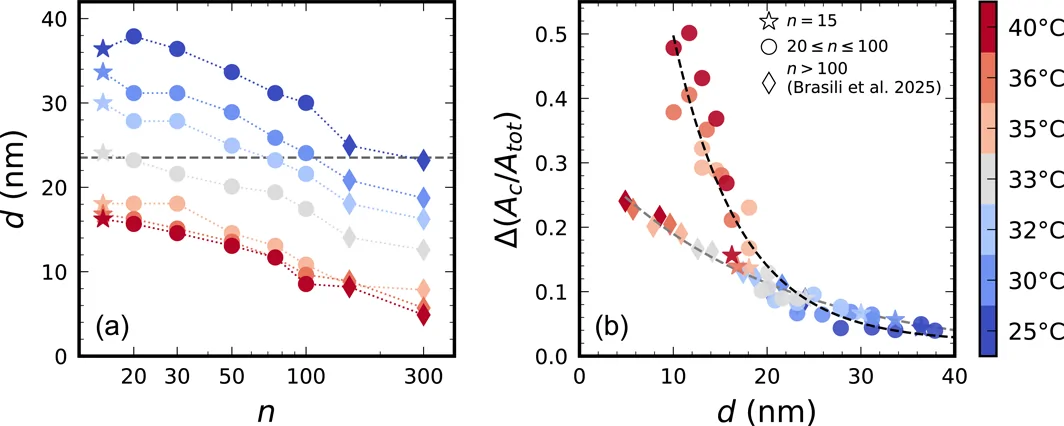
(a) Surface‐to‐surface distance d between NPs as a function of n for different temperatures, derived from structure factors of NPs incorporated in the microgel corona; the horizontal dashed line (d=23.7 nm) marks the intersection between the exponential fits in panel b. (b) Degree of plasmon coupling $\Delta \left(A_{C}/A_{\mathrm{tot}}\right)$ as a function of d; dashed lines are the fits to exponential decay.

Following the model in Jain et al. [34], we fit separately each branch with an exponential decay $\Delta \left(A_{C}/A_{\mathrm{tot}}\right)=C\;\exp\left(-d/l_{d}\right)$, where $l_{d}$ is the characteristic decay length, quantifying the attenuation of the near field coupling, and C represents an effective coupling strength. We find C=0.3 and $l_{d}=19.1$ nm for the lower branch, whereas C=1.9 and $l_{d}=7.2$ nm for the higher one, revealing the onset of two distinct mechanisms governing plasmon coupling. The second one, that is prevalent at intermediate n, leads to stronger coupling but at the same time has a more rapid decay as a function of d. In this respect, we must consider that plasmon coupling in NP assemblies also depends on other parameters than the interparticle distance d, including the overall size of the assemblies and their spatial arrangement, which can vary from compact to highly ramified structures and is often characterized in terms of branching and fractal dimension [31, 35, 36]. These features can be affected by the aggregation of microgel–NP complexes that would bring NPs confined to different microgels in close proximity, resulting in larger NP clusters. However, d represents the nanoscale distance between nearest‐neighbor NPs, without distinguishing whether they belong to the same microgel or two approaching microgels. Assessing the colloidal stability of microgel–NP complexes requires probing larger length scales (microscopic range), which are not fully accessible in our SAXS measurements. Therefore, in the following paragraph, we report a dynamic light scattering (DLS) investigation of the microgel–NP complexes aimed at constructing their phase diagram to gain further insights on the two coupling mechanisms and to link them to the re‐entrant behavior of plasmon coupling.

### 2.3 Re‐Entrant Condensation of Microgel–NP Complexes Drives Plasmon Coupling

We interpret the re‐entrant optical behavior of the microgel–NP complexes within the context of the colloidal stability of charged particles co‐dispersed with oppositely charged polyelectrolytes. The electrostatic adsorption of polyelectrolytes leads to heterogeneous charge distribution on the primary particle's surface, resulting in charge‐patched colloids and introducing an attractive contribution to the effective interparticle interactions. This affects the colloidal stability and promotes the formation of stable aggregates, whose finite size increases with the concentration of the adsorbing species [27]. Moreover, due to the strong electrostatic correlations between the adsorbed polyelectrolytes and the charge fractionalization mechanism [37], it is possible that polyelectrolytes adsorb in excess with respect to the amount required for complete neutralization of the surface charge, resulting in overcharging, that is, the inversion of the net charge sign of the complex relative to the primary particles. As a consequence, re‐entrant condensation occurs: beyond a certain polyelectrolyte loading, the aggregate size reaches a maximum and then decreases upon further adsorption. This phenomenology has been extensively studied in systems, where liposomes [27, 38] or plasmonic NPs [30, 31] act as primary colloids, and has more recently been reported also for microgels [28, 29]. In particular, in the case of thermoresponsive microgels, aggregation phenomena can be further modulated by temperature, as the collapse of the polymer network across the VPT leads to a substantial increase in their surface charge density. Consequently, surface charge heterogeneities, responsible for the attractive contribution in the pair interaction potential, can increase significantly. As a result, the effective interparticle interactions become strongly temperature‐dependent, adding an additional level of complexity to the aggregation behavior. This effect has been reported in Sennato et al. [29], where a similar phenomenology was observed when the adsorbing species were silica NPs rather than charged macromolecules. In the case of plasmonic NPs, such temperature‐driven modulation of patchy interactions is expected to introduce new interaction regimes, with direct consequences on NP organization and on the resulting plasmon coupling.

Here, we analyze the thermally induced aggregation of microgel‐NPs systems for different values of n, and relate it to their optical properties. In Figure 5a,b, we report the trends of hydrodynamic radius $R_{H}$ and electrophoretic mobility $\mu_{e}$ as a function of n for a range of temperatures across $T_{\text{VPT}}$. Below $T_{\text{VPT}}$, the values of $R_{H}$ are always smaller than those of bare microgels, demonstrating the colloidal stability of the complexes over the entire range of n investigated. Moreover, the values of $R_{H}$ decrease with n, particularly for n=15, consistent with the NP‐induced shrinking of the microgels [26]. As temperature increases, $R_{H}$ decreases following the VPT, until—at intermediate values of n (20≤n≤100)—it abruptly increases, exceeding the size of individual microgel–NP complexes measured at 25°C. This indicates the formation of aggregates, consistent with the charge‐patch aggregation phenomenon. The size of the aggregates increases with n up to a maximum around n=50 and then decreases until the colloidal stability of the primary colloids (microgel–NP complexes) is restored for n>100. Examining the behavior of $\mu_{e}$, we observe that the microgels are initially positively charged due to the amine groups from the initiator of the chemical synthesis. The charge variations become more evident at higher temperatures because the surface charge density grows with the compaction of the surface area and because the mobility is enhanced by the reduced particle size. As n increases, the adsorption of anionic NPs lowers the net charge of the complexes until charge inversion occurs, between n=20 and n=30, depending on temperature. Beyond the isoelectric point, the surface charge density becomes progressively more negative and inhomogeneous, and this is what drives the re‐entrant condensation. Importantly, throughout this process, the surface coverage by adsorbed NPs remains low, even in the collapsed state (θ<25% for n≤100, see Supporting Information S1: Section S2 and Figure 5c), so that the microgel surface never becomes uniformly charged and the charge‐patch description holds over the entire range explored. Considering the surface coverage at charge inversion, a simple geometrical argument shows that the NP‐covered regions are far more densely charged than the bare microgel surface. This strong charge asymmetry explains why the maximum aggregate size occurs at a loading well beyond that of net‐charge neutrality (see Supporting Information S1: Section S2).

**FIGURE 5:**
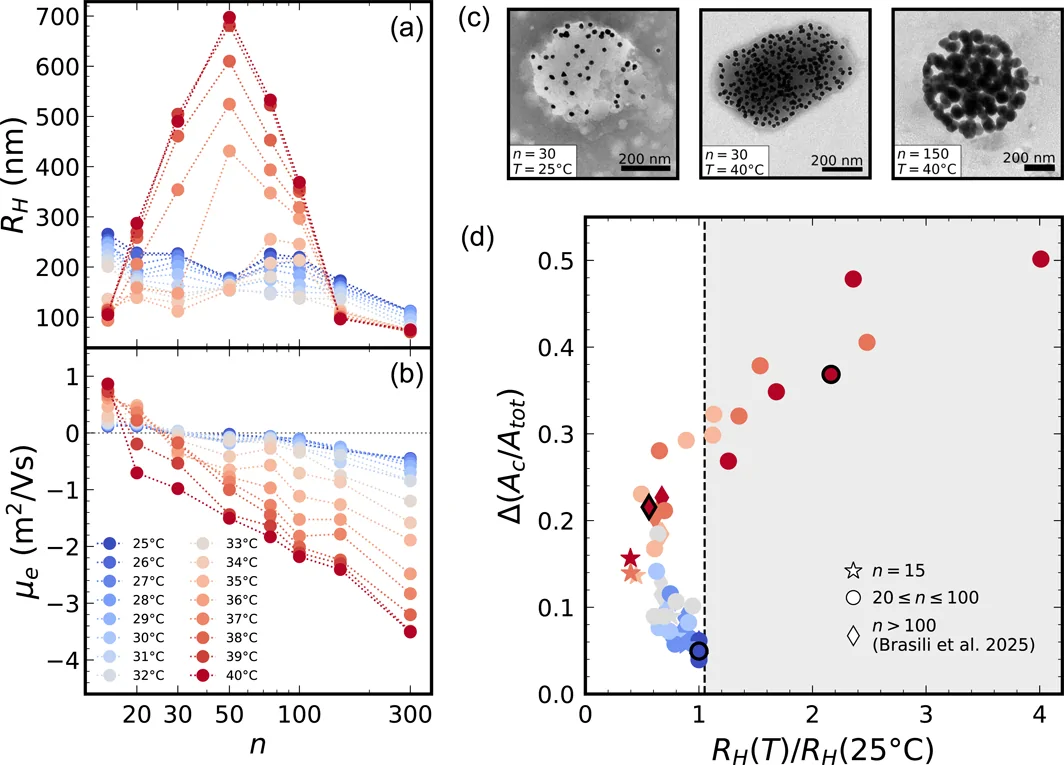
Hydrodynamic radius $R_{H}$ (a) and electrophoretic mobility $\mu_{e}$ (b) of the microgel–NP complexes as a function of n, for different temperatures across the VPT. (c) Representative electron microscopy images of the samples in selected (n,T) conditions: individual complexes for (30, 25°C) and (150, 40°C), and aggregated structures, for (30, 40°C). (d) Degree of plasmon coupling $\Delta \left(A_{C}/A_{\mathrm{tot}}\right)$ as a function of $R_{H}\left(n,T\right)/R_{H}\left(n,25^{◦}C\right)$; the vertical dashed line divides the region of colloidal stability from that of complex aggregation (shaded in gray); the data points highlighted by black edges correspond to the images in Panel c.

It is worth noting that thermally induced aggregation occurs over the same range of n corresponding to the stronger‐coupling branch in Figure 4b. To quantitatively investigate this aspect, we plot the plasmon coupling obtained from extinction spectra in different experimental conditions (n,T) as a function of the ratio $R_{H}\left(n,T\right)/R_{H}\left(n,25^{◦}C\right)$ measured under the same conditions (Figure 5d). We find that all data collapse onto a common trend, with $\Delta \left(A_{C}/A_{\mathrm{tot}}\right)$ increasing both when the microgel VPT induces particle shrinking without compromising colloidal stability $R_{H}\left(n,T\right)/R_{H}\left(n,25^{◦}C\right)\leq 1$, and, even more markedly, when the complexes aggregate (gray‐shaded region). One might wonder whether the growth of extinction at λ>570 nm, and hence the re‐entrant behavior of $\Delta \left(A_{C}/A_{\mathrm{tot}}\right)$, could be attributed to scattering from the large aggregates that form under the same experimental conditions. This hypothesis is ruled out qualitatively by the concurrent redshift of $\lambda_{\text{LSPR}}$, which mirrors the behavior of $\Delta \left(A_{C}/A_{\mathrm{tot}}\right)$ (Figure 2): a featureless scattering background cannot produce such redshift, which is instead a specific signature of interparticle plasmon coupling. We further substantiate this conclusion quantitatively in Supporting Information S1: Section S3, where we correct the extinction spectra by subtracting the contribution of size‐matched NP‐free microgel aggregates. This analysis shows that aggregate scattering leads at most to a slight underestimate of the coupling degree, leaving the re‐entrant dependence on n and the overall picture presented here unaffected.

These results provide the first direct evidence of a relationship between the plasmon coupling of NPs adsorbed to thermoresponsive microgels and the colloidal stability of the microgel–NP complexes. Moreover, by using the same markers of Figure 4b, we set a correspondence with the two branches of that plot. It then becomes evident that the data points belonging to the strong‐coupling branch coincide with the onset of aggregation, demonstrating that the formation of NP assemblies extended across the corona of approaching microgels is responsible for the enhanced plasmon coupling observed, beyond that induced by the increasing temperature in isolated complexes. Indeed, in the case of stable suspensions, plasmon coupling originates from interactions among neighboring NPs confined within the spherical corona of individual microgels [15]. In contrast, upon aggregation of the microgel–NP complexes, the NP assemblies responsible for coupling extend across the coronas of two or more adjacent microgels, thereby involving a larger number of NPs and effectively generating two interacting NP layers at each contact surface. This configuration, reminiscent of that reported for superimposed planar NP layers [39], results in a substantially amplified coupling strength. The electron microscopy images of Figure 5c further support this interpretation, providing direct visualization of isolated complexes, for the samples (30, 25°C) and (150, 40°C), and aggregated structures, for the sample (30, 40°C), where NPs can be clearly observed in contact across the coronas of neighboring microgels.

Building on this evidence, we establish a direct correspondence between the phase diagram of the colloidal dispersion and the optical response of the system. In Figure 6, we delineate the regions of the n−T plane where the complexes are colloidally stable from those where they aggregate (gray area). The aggregation region coincides with the regime of strongest coupling identified in Figure 4b for intermediate n. Moreover, the shorter decay length found for aggregating complexes is consistent with previous results obtained for interacting planar layers of NPs [40]. Each experimental condition (n,T) investigated by extinction spectroscopy is then mapped onto the same phase diagram. We assigned colors based on the magnitude of $\Delta \left(A_{C}/A_{\mathrm{tot}}\right)$ on the right scale that is mapped on the corresponding LSPR peak wavelength $\lambda_{\text{LSPR}}$, assigning to each wavelength its corresponding spectral color. We further highlight the points corresponding to the stronger‐coupling branch using circles with a black edge. This diagram clearly shows that the aggregation of the complexes is precisely what determines a more intense plasmon coupling compared to that observed as a function of temperature for individual complexes. The onset of aggregation thus marks a distinct optical regime characterized by a pronounced enhancement of plasmon coupling besides that observed for isolated complexes upon temperature increase.

**FIGURE 6:**
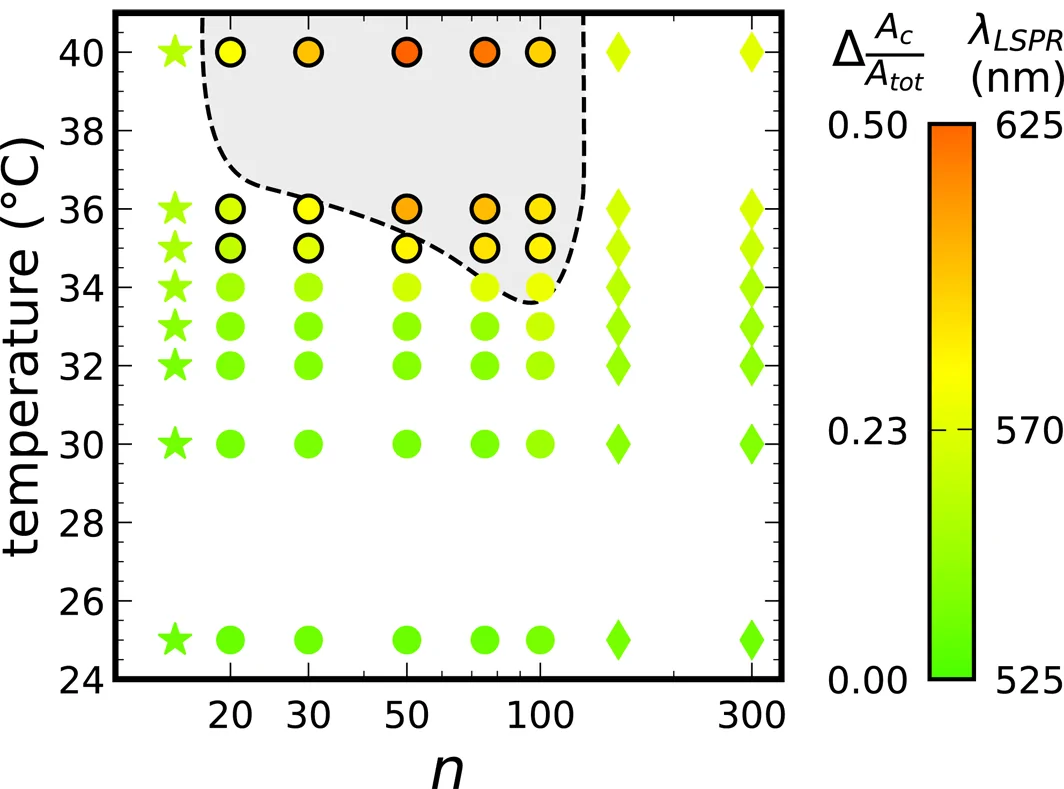
Phase diagram of microgel–NPs in the n−T plane: the dashed black line delineates the boundary between the colloidal stability of the complexes and the aggregation region (shaded in gray), as identified from DLS measurements by applying the threshold $R_{H}\left(n,T\right)>R_{H}\left(n,25^{◦}C\right)$, to define the aggregation onset; the colored points denote the samples for which extinction spectra were collected: we used the same markers of Figure 4b to set a correspondence with the two branches of that plot (circles with black edges identify the high‐coupling branch), whereas the color is assigned based on values of $\Delta \left(A_{C}/A_{\mathrm{tot}}\right)$, according to the colormap on the right; the colormap encodes the LSPR wavelength extracted from the extinction spectra, providing a direct visualization of the optical properties of the system.

We finally note that the re‐entrant plasmonic response reported here is not fully reversible under heating–cooling cycle: for samples that aggregate, both colloidal structure and optical properties persist in the high‐coupling state upon cooling; for nonaggregating samples, the VPT is fully reversible and the optical response is largely recovered, with a small residual offset at 25°C. A detailed analysis of the thermal reversibility is reported in Supporting Information S1: Section S4.

Overall, these findings establish a unified framework linking thermally driven phase behavior of microgel–NP complexes to the collective plasmonic response, providing a powerful approach to rationally design responsive plasmonic materials through the controlled modulation of colloidal interactions.

## 3 Conclusions

Microgel–NP complexes represent a versatile class of plasmonic colloidal systems in which plasmon coupling can be dynamically modulated by exploiting the intrinsic responsiveness of microgels to external stimuli, making them highly relevant for the development of novel soft photonic materials. In this work, we used electrostatically assembled complexes to investigate their colloidal stability, an aspect that has received limited attention so far, despite its marked impact on the optical response of this hybrid system. We revealed a surprising re‐entrant behavior of the plasmon coupling as a function of the NP‐to‐microgel number ratio n. By combining SAXS measurements of NP–NP spatial correlations with extinction spectroscopy, we rationalized the mechanisms underlying this phenomenology. To this aim, we established a quantitative relationship between plasmon coupling and the surface‐to‐surface distance d between NPs adsorbed to microgels. Intriguingly, we found that this relationship is not universal; instead, it branches in two distinct behaviors depending on the NP loading. At comparable interparticle distances, the plasmon coupling is significantly enhanced for intermediate surface coverages (20≤n≤100), whereas for both low (n=15) and high (n>100) loadings, it follows the same trend as a function of d and remains systematically weaker with respect to intermediate n values.

We then demonstrated that this dual behavior originates from the aggregation of microgel–NP complexes occurring only for intermediate n, as revealed by DLS measurements. In this regime, partial NP coverage results in oppositely charged patches on the surface of the complexes, promoting the formation of stable, finite‐size clusters. Upon further increasing NP coverage, charge uniformity is progressively restored (with overcharging), electrostatic repulsion dominates, and the complexes regain colloidal stability. A clear correspondence is found between the aggregation phase‐diagram and the deviation of the plasmon coupling from the trend of isolated microgel–NP complexes.

On this basis, we propose that the splitting of the coupling‐distance trends arises from the existence of two distinct plasmon coupling regimes. In colloidally stable microgel–NP complexes, coupling is governed primarily by NP organization within the corona of individual microgels. In contrast, when the complexes aggregate, additional coupling emerges between NPs belonging to neighboring microgels, giving rise to a marked overall enhancement. This picture naturally explains the observed re‐entrant behavior of the optical properties: when aggregation is suppressed, the coupling intensity decreases again.

Looking forward, we believe that the thermal reversibility of the plasmonic response, which we have shown to be largely recovered in the region of colloidal stability represents an important benchmark for designing fully reversible optical materials. Moreover, extending this study beyond the present case, it will be very interesting in the future to see whether these two coupling regimes also hold in different experimental conditions, varying, for example, the architecture of the polymer network through the fraction of crosslinker monomers, or NP size. In addition, the present case deals with microgel charges originating solely from the initiator that are uniformly positive and localized in the outer corona, but there is the need to also explore how different spatial distributions of charges and the presence of oppositely charged groups within the polymer network affect NP adsorption and plasmon coupling.

## 4 Experimental

### 4.1 Microgel Synthesis

Cationic microgels are synthesized by surfactant‐free radical polymerization as detailed previously [28], using NIPAM monomers (Sigma‐Aldrich, MW = 113.16 Da), 5% molar fraction of N,N′‐Methylenebis(acrylamide) (BIS, Sigma‐Aldrich, MW = 154.17 Da) as a crosslinker and 1% molar fraction of 2,2′‐Azobis(2‐methylpropionamidine) dihydrochloride (AIBA, Sigma‐Aldrich, MW = 271.19 Da) as ionic initiator. Briefly, we dissolve 1.25 g NIPAM and 92 mg BIS in 148 mL of deionized water. Separately, 30 mg AIBA are dissolved in 2 mL of deionized water. The solution with the reagents is bubbled with argon for 30 minutes, and we then heat it up to 70°C and add the initiator to start the reaction. After 6 h, the microgel dispersion obtained is cooled to room temperature and filtered through glass wool. To prevent bacterial growth, we add 2 mM NaN_3_. The obtained microgels have a hydrodynamic radius $R_{H}=$ 286 nm and number density $n_{mg}^{\left(0\right)}=1.63\times 10^{12}$ mL^−1^ (volume fraction φ=0.16) at 20°C, as determined by DLS and viscosimetry measurements.

### 4.2 Preparation of Microgel–Nanoparticle Samples

To prepare microgel–NP samples, we use spherical gold NPs (Ted Pella) with nominal number density $n_{\text{NP}}^{\left(0\right)}=7.0\times 10^{11}$ ${\text{mL}}^{-1}$. NPs are stabilized by a citrate capping that provides them with a negative charge, resulting in the electrophoretic mobility $\mu_{e}=-3.42\times 10^{-8}$ $m^{2}$/Vs and have a radius a=9.3 nm, as previously evaluated [15, 26]. The microgel dispersion is diluted 250 times in 0.2 mM NaN_3_ to prevent bacterial growth. We verified by DLS measurements that this salt concentration is low enough to exclude any effect of ionic strength on the colloidal stability of the microgels or NPs. The NP dispersion is then either diluted with MilliQ water or concentrated by centrifugation to obtain the desired NP number densities for each sample. Finally, equal volumes of the microgel and NP dispersions are mixed to obtain samples with fixed $n_{mg}=3.26\times 10^{9}$ ${\text{mL}}^{-1}$ and varying NP‐to‐microgel number ratio $n=n_{\text{NP}}/n_{mg}$.

### 4.3 Extinction Spectroscopy

Extinction spectra in the UV–Visible‐NIR spectral range are acquired with a resolution of 0.1 nm, using a v‐570 double ray spectrophotometer (Jasco, IT) equipped with a Peltier thermostated holder EHC‐505. We measure the spectra of the samples at varying temperature between 20°C and 40°C; after every temperature change, samples are kept thermalizing for at least 5 minutes and then spectra are acquired. All the spectra reported in the figures are normalized to the extinction at 400 nm, which corresponds to the gold interband transitions and is unaffected by the particle size, shape, and environment [41]. To quantify the modifications of the LSPR at varying temperature across microgel VPT, we use the wavelength $\lambda_{\text{LSPR}}$ of its maximum and the coupling degree, defined as the spectral weight of the region between 570 and 800 nm [15], where coupled plasmon modes prevalently contribute to the extinction:

(1) $$\Delta \frac{A_{C}}{A_{\mathrm{tot}}}=\frac{A_{C}\left(T\right)}{A_{\mathrm{tot}}\left(T\right)}-\frac{A_{C}^{\left(NP\right)}}{A_{\mathrm{tot}}^{\left(NP\right)}},$$

where A(T) indicates the area underlying the spectrum acquired at temperature T; the pedices tot and C refer to the overall spectral range and the region of coupled modes, respectively; $A_{\mathrm{tot}}^{\left(NP\right)}$ and $A_{C}^{\left(NP\right)}$ are the areas computed in the same regions on the reference spectrum of noninteracting NPs.

### 4.4 Small Angle X‐Ray Scattering

SAXS experiments are performed on samples filled in capillaries of 1.5 mm diameter and placed at the 3‐m sample‐to‐detector distance. The exposure time for acquisitions is set to 1 s and 14 scattering patterns are acquired for each sample. Scattering patterns are recorded at 12 keV using a two‐dimensional EigerX 4‐M detector (Dectris, Baden, Switzerland). This allows measurements in the range of q‐vector between 0.002 and 0.38 Å^−1^, where q is defined as q=(4π⁄λ)sinθ, 2θ is the scattering angle, and λ is the wavelength of the radiation. Scattering patterns of an empty capillary and of a capillary filled with water are recorded for the normalization of the intensity to absolute units and background subtraction, respectively. Experiments are conducted at selected temperatures between 25°C and 40°C, employing a Huber Ministat 125 thermostat. After each temperature change, samples are left to equilibrate for 5 minutes before measurements. The processing and averaging of the scattering patterns are performed by the software Foxtrot (SOLEIL software group and SWING beamline).

For a collection of particles, the scattered intensity I(q) can be expressed in terms of the form factor P(q) of single particles and of the structure factor S(q) of the system as $I\left(q\right)=Nv^{2}\Delta \rho^{2}P\left(q\right)S\left(q\right)$, where N and v are the number density and the volume of the scattering particles, and Δρ is the contrast in electron density ρ between particles and solvent. S(q) is the interference introduced by interparticle correlations and can be expressed in terms of the Fourier transform of the pair distribution function g(r) as

(2) $$S\left(q\right)=1+\rho \int_{V}g\left(r\right)e^{-i\vec{q}\cdot \vec{r}}d\vec{r}.$$

Since for a dilute system of noninteracting particles S(q)=1, we measure the form factor P(q) of gold NPs on the stock solution [31]. In microgel–NP samples, the much higher electron density of gold relative to the polymer matrix ensures that, for the chosen acquisition times, the measured scattered intensity I(q) originates exclusively from the NPs [33]. The NP structure factor S(q) is therefore obtained, for each sample, as S(q)=I(q)/P(q). We analyze the S(q) to extract the average surface‐to‐surface distance d between the nearest neighbor NPs. For microgel–NP complexes, two distinct q regions can be identified, each one associated with structural correlations at different length scales [15]. At low q (approximately below 0.1 ${\text{nm}}^{-1}$), the scattering captures microscopic features of the NP arrangement, for example, reflecting the confinement of NPs within a spherical shell region in the microgel corona. At high q (above 0.1 ${\text{nm}}^{-1}$), the range of interest here, the scattering probes nanoscale features of the local NP packing. We focus on the first peak in this high‐q range, from which we calculate the interparticle distance as

(3) $$d\left(n,T\right)=2\pi /q_{p}\left(n,T\right)-2a,$$

where $q_{p}\left(n,T\right)$ is the position of the peak for the sample (n,T). We note that this peak identifies the dominant nearest‐neighbor NP distance, irrespective of whether they are adsorbed to the same microgel or located on the surfaces of two approaching microgels.

### 4.5 Hydrodynamic Radius and Electrophoretic Mobility Measurements

Hydrodynamic radius $R_{H}$ and electrophoretic mobility $\mu_{e}$ of the suspended microgels are measured almost simultaneously by DLS and electrophoretic light scattering, respectively. We employed a NanoZetaSizer apparatus (Malvern Instruments LTD) equipped with a He–Ne laser (5 mW power, 633 nm wavelength). DLS intensity autocorrelation functions are measured at an angle of 173°, in quasi‐backscattering geometry. The correlograms are analyzed by means of the CONTIN algorithm [42] to obtain the intensity weighted distributions of $R_{H}$. The electrophoretic mobility is obtained by phase analysis light scattering [43], which allows accurate measurements of low‐mobility samples. For soft, solvent‐permeable colloids such as pNIPAM microgels in both swollen and collapsed states [44], $\mu_{e}$ cannot be rigorously converted into a ζ‐potential or a surface charge density, since no sharp slipping plane exists when the flow penetrates the charged polymer network [45]. We therefore report and discuss the $\mu_{e}$ itself, which provides a robust, model‐independent indicator of the net charge sign and its evolution with n.

Temperature trends are measured using ascending ramps between 25°C and 40°C with steps of 1°C. At each step, the sample is allowed to equilibrate for 5 minutes at the target temperature before performing measurements. The reported values of $R_{H}$ or $\mu_{e}$ and the associated errors are the mean and standard deviation of at least three independent measurements.

### 4.6 Electron Microscopy

Transmission electron microscopy measurements were performed using a Tecnai G2 12 TWIN (Thermo Fisher Scientific) setup, operating at 120 kV and equipped with an electron energy loss filter (Biofilter, Gatan Inc.) and a slow‐scan charge‐coupled device camera (794 IF, Gatan Inc.). All the samples have been prepared by depositing 20 μl of microgel–NP dispersion on a 300 mesh copper grid covered by a thin amorphous carbon film. Following the protocol already used in a previous investigation [28], samples have been deposited both at room temperature and at 40°C. The samples deposited at 25°C were stained by phosphotungstic acid by adding 10 μl of 2% aqueous solution (with pH adjusted to 7.3 using 1 N NaOH) to enhance image contrast and evidence the contour of microgels.

## Author Contributions

**Angela Capocefalo:** investigation, writing – original draft, conceptualization, data curation, formal analysis, methodology, visualization. **Francesco Brasili:** conceptualization, investigation, writing – original draft, formal analysis, data curation, methodology, visualization. **Javier Pérez:** investigation. **Edouard Chauveau:** investigation. **Stefano Casciardi:** investigation, writing – review and editing. **Andrea Militello:** validation, writing – review and editing. **Francesco Sciortino:** writing – review and editing, validation, resources. **Emanuela Zaccarelli:** writing – review and editing, resources, validation, methodology, funding acquisition. **Federico Bordi:** writing – review and editing, resources, validation. **Domenico Truzzolillo:** writing – review and editing, validation, conceptualization, supervision, funding acquisition, methodology, resources, project administration. **Simona Sennato:** supervision, conceptualization, funding acquisition, validation, writing – review and editing, methodology, resources, project administration.

## Conflicts of Interest

The authors declare no conflicts of interest.

## Supporting information


Supporting Information S1

## Data Availability

The data that support the findings of this study are openly available in Zenodo at https://doi.org/10.5281/zenodo.20119925.
