# Supplementary material for: Phase Behavior of Thermoresponsive Colloids Drives Re‐Entrant Plasmon Coupling
Source: Nanophotonics. 2026 Nov 13;15(22):e70322. doi: 10.1002/nap2.70322 (PMC13768810; doi:10.1002/nap2.70322)
Supplement: Supplementary file 1 [file NAP2-15-e70322-s001.pdf]

Supporting Information for: Phase behavior of thermoresponsive colloids drives re-entrant plasmon coupling

Angela Capocefalo,[a,b] Francesco Brasili,*[a,b] Javier Pérez,[c] Edouard Chauveau,[d] Stefano Casciardi,[e] Andrea Militello,[e] Francesco Sciortino,[b] Emanuela Zaccarelli,[a,b] Federico Bordi,[b] Domenico Truzzolillo,*[d] and Simona Sennato[a,b]

[a] *Institute for Complex Systems, National Research Council, Piazzale Aldo Moro 5, 00185, Roma, Italy*

[b] *Physics Department, Sapienza University of Rome, Piazzale Aldo Moro 5, 00185, Roma, Italy*

[c] *Synchrotron SOLEIL, L'Orme des Merisiers, Départementale 128, 91190, Saint-Aubin, France*

[d] *Laboratoire Charles Coulomb, CNRS–Université de Montpellier, UMR 5221, 34095, Montpellier, France*

[e] *Dipartimento di medicina, epidemiologia, igiene del lavoro e ambientale, Istituto Nazionale per l'Assicurazione contro gli Infortuni sul Lavoro, Via Fontana Candida 1, 00078, Monteporzio Catone, Roma, Italy*

E-mail: francesco.brasili@cnr.it; domenico.truzzolillo@umontpellier.fr

D.T. and S.S. contributed equally to this work.

S1. Additional extinction spectroscopy and SAXS data

Reference extinction spectra of the two components of the hybrid microgel-NPs complexes, namely gold nanoparticles (NPs, 20 nm diameter) and pristine microgel dispersions, are shown in Figs. S1a and S1b, respectively, at different temperatures across the microgel volume phase transition (VPT). The sample numbers densities are $n_{\mathrm{NP}} = 4.89 \times 10^{11}$ mL^{-1} and $n_{mg} = 3.26 \times 10^{9}$ mL^{-1} for NPs and microgels, respectively, the same of the microgel–NPs sample with the NP-to-microgel number ratio $n = 150$. To directly compare the two datasets, the spectra are reported in absolute extinction units rather than being normalized to the extinction at 400 nm, using the same scale. The NP spectrum exhibits the characteristic localized surface plasmon resonance (LSPR) peak at $\lambda_{\mathrm{LSPR}} = 525$ nm and remains unchanged between 25°C and 40°C. In contrast, the microgel spectra evolve with temperature as the particles collapse, becoming smaller and denser across the VPT, leading to an increasing turbidity.[1] Comparison of the two spectra shows that, even above the VPT where the microgel contribution is largest, the maximum extinction of the pristine microgel dispersion remains at least fivefold smaller than the LSPR peak of the NP dispersion. The contribution of the microgels to the extinction spectra of the hybrid complexes can therefore be considered

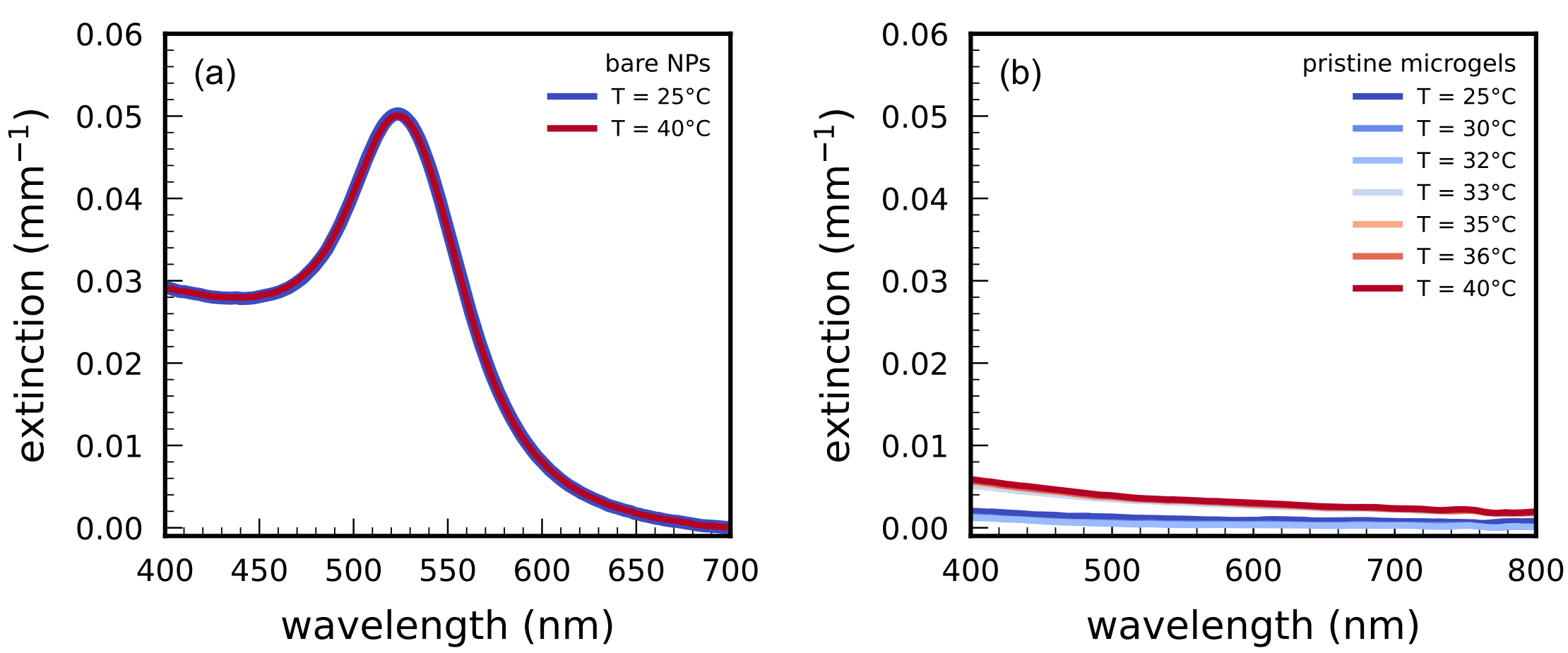

Figure S1 (a) Extinction spectra of stock NPs, at the same NP concentration of the microgel-NPs sample with $n = 150$, at 25°C and 40°C. (b) Extinction spectra of pristine microgels at varying temperature across the VPT.

negligible. Moreover, even in the lowest-n samples, where the microgel contribution may become more appreciable, its monotonically decreasing spectral profile would only lead to a slight underestimation of the LSPR shift $\Delta\lambda_{\rm LSPR}$ and of the coupling degree $\Delta(A_C/A_{tot})$ at high temperatures. A thorough analysis of these aspects is reported in Section S3.

Additional extinction spectra and the structure factors $S(q)$ of the microgel–NPs samples are reported in Fig. S2 and Fig. S3, respectively, Specifically, we show in the figures the datasets at varying temperature for $n = 20$ and $n = 75$ samples. The corresponding datasets for the $n = 150$ sample were previously published in ref. 2 and are also available through the Zenodo repository (doi:10.5281/zenodo.20042415).

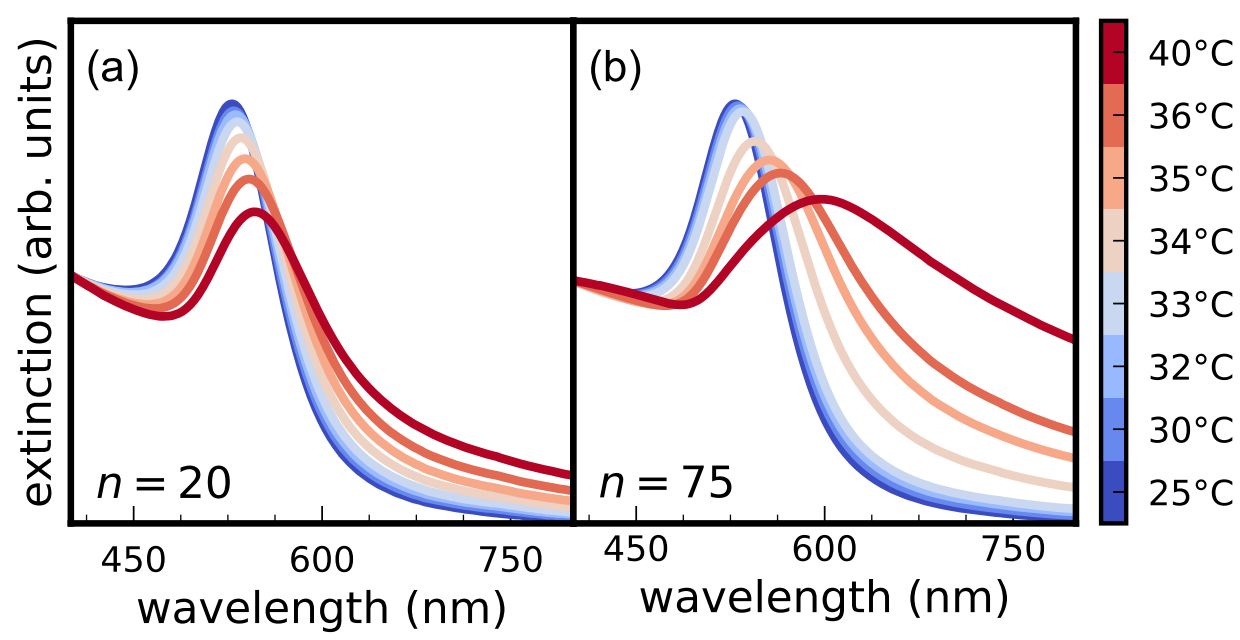

Figure S2 Extinction spectra of microgel-NPs at different temperatures for $n = 20$ (a) and $n = 75$ (b).

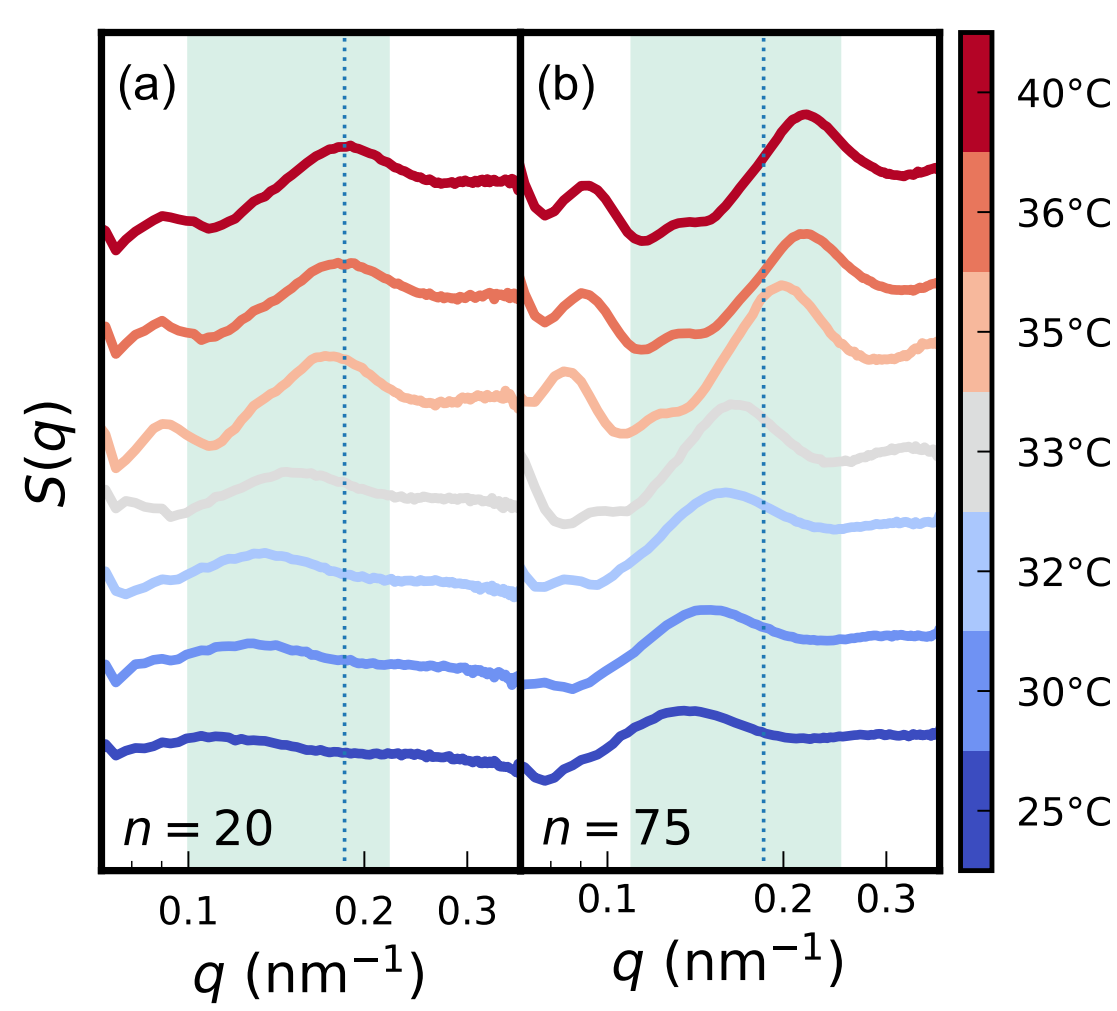

Figure S3 Structure factors of the microgel-NPs samples at different temperatures for $n = 20$ (a) and $n = 75$ (b). The light green color highlights the peaks corresponding to the distance between NPs.

S2. Charge-patch asymmetry accounts for the n-offset between charge inversion and aggregation maximum

The electrophoretic mobility μ_e of the microgel-NPs complexes (Fig. 5b) provides a robust, model-independent measurement of the sign of the net charge and of the loading n at which it reverses. We note, however, that electro-osmotic contributions to the electrophoretic force can shift the mobility reversal with respect to the point of zero net charge, particularly when ion-ion correlations in the Debye layer are strong[3,4]. Since ion-ion correlations are enhanced by high adsorption energies – which are expected to be larger for the collapsed microgels due to their higher surface charge density – the condition $\mu_e = 0$ is a more reliable marker for the isoelectric point at low temperature, where the microgels are swollen and correlations are weaker. We therefore use the charge inversion observed at 25°C, $n \simeq 30$, as our best estimate of the charge inversion. Based on this, we derive an order-of-magnitude estimate of the charge inhomogeneity between NP-covered and bare regions of the particles' surface. We model the surface of a complex as composed of two types of patches: a fraction θ covered by adsorbed NPs, carrying an effective negative charge density $-\sigma_{\mathrm{NP}}$, and the complementary bare-microgel fraction $1-\theta$, carrying a positive charge density $+\sigma_{\mu\mathrm{gel}}$. Within this two-patch description, θ can be evaluated as the projected-area coverage of the n_{ads} NPs adsorbed to the microgel as:

$$\theta = n_{ads} \frac{\pi a^2}{4\pi R_{\mu\mathrm{gel}}^2} \quad ,$$

where $R_{\mu\mathrm{gel}}$ is the radius of the adsorbing microgel and $a = 9.3$ nm is the NP radius, and the mean net charge density is given by:

$$< \sigma > = (1-\theta)\, \sigma_{\mu\mathrm{gel}} - \theta\, \sigma_{\mathrm{NP}} \quad ,$$

which vanishes at a coverage $\theta_0 = \frac{\sigma_{\mu\mathrm{gel}}}{\sigma_{\mathrm{NP}}+\sigma_{\mu\mathrm{gel}}}$, so that:

$$\frac{\sigma_{\mathrm{NP}}}{\sigma_{\mu\mathrm{gel}}} = \frac{1-\theta_0}{\theta_0} \quad .$$

We now focus on the microgel at $T = 40°\mathrm{C}$ ($R_{\mu\mathrm{gel}} \approx 100 \div 105$ nm), since aggregation occurs only in the collapsed state. As established in ref. 5 for less charged microgels and thus still valid for the slightly more charged microgels used here, for $n = 30$ essentially all NPs are adsorbed to the microgels ($n_{ads} \simeq n$) and, pushed outwards during microgel collapse without significant desorption, at 40°C they are all located on the external surface of the hybrid complex[2]. We can therefore estimate $\theta_0 \approx 6\%$ and, consequently, $\sigma_{\mathrm{NP}}/\sigma_{\mu\mathrm{gel}} \approx 15$.

Although this estimate gives only an order of magnitude and rests on simplifying assumptions (two discrete patch populations, uniform charge densities within each, and a projected-area coverage), it clearly highlights that charge reversal is achieved at a very low surface coverage (see also electron microscopy in Fig. 5d), implying that the NP-covered patches are far more densely charged than the bare microgel surface. Consistently, the coverage remains low over the entire range of n in which aggregation occurs ($\theta < 25\%$ for $n \leq 100$), so that the surface never becomes uniformly charged and the system always resides in the charge-patch regime. The strong asymmetry $\sigma_{\mathrm{NP}} >> \sigma_{\mu\mathrm{gel}}$ also clarifies why the maximum aggregate size is reached at a loading well beyond the charge-inversion point (compare Figs. 5a and 5b). Because the NP-covered patches are much more densely charged than the bare microgel surface, net charge neutrality is reached at a very low coverage. In contrast, the attractive contribution to interactions arising from charge inhomogeneity scales geometrically as $\theta(1-\theta)^6$, becoming stronger with n well past θ_0. The aggregation maximum is therefore shifted to higher loadings respect to net neutrality, where the inhomogeneity is substantial while the (now overcharged) average surface charge density is still moderate; at higher n, the growing surface charge restabilizes the complexes through electrostatic repulsion between facing portions of the surfaces of approaching particles. Had the two patch densities been comparable ($\sigma_{\mathrm{NP}} \approx \sigma_{\mu\mathrm{gel}}$), net neutrality and maximum inhomogeneity would both fall near $\theta = 0.5$, and the two features would nearly coincide.

S3. Effect of microgel collapse and aggregation on the extinction spectra

As discussed in the main text, the microgel–NP suspensions may contain mesoscale aggregates at temperatures above the microgel VPT, depending on the NP-to-microgel number ratio n (Fig. 5). Because large colloidal aggregates scatter light with higher intensity relative to a non-aggregated dispersion, we assessed whether this contribution could partly account for the observed modifications of the extinction spectra and the apparent increase in the coupling degree $\Delta(A_C/A_{tot})$.

To obtain a reference for the scattering background, we induced aggregation in pristine (NP-free) microgel dispersions by adding NaCl at concentrations between 5 and 50 mM and equilibrating at 40°C, where aggregation, and hence the potential scattering artefact, is most pronounced. In all reference samples, the microgel number concentration was kept identical to that of the corresponding microgel–NP suspensions, so that differences in the extinction spectra reflect only the presence of the NPs and their plasmonic response. DLS measurements confirmed that this procedure yields aggregates with hydrodynamic radii spanning the range observed in the microgel–NP samples. For each value of n, we identified the NP-free aggregate sample with the closest matching R_H and used its extinction spectrum as a proxy for the non-plasmonic scattering background. For the three values of n at which the microgel–NP suspensions do not exhibit aggregation ($n = 15$, $n = 150$ and $n = 300$), the reference spectrum was taken as that of the pristine microgel dispersion at 40°C in the absence of added salt (Fig. S1). Fig. S4 shows, for each value of n investigated, three spectra: (i) the extinction spectrum of the microgel–NP suspension at 40°C, (ii) the extinction spectrum of the size-matched NP-free microgel aggregate, and (iii) the corrected spectrum obtained by subtracting (ii) from (i). Unlike the spectra shown in the main text, which are normalized to the extinction at 400 nm, here all spectra are reported in absolute extinction units, since the background subtraction requires that the two spectra being compared are on the same scale.

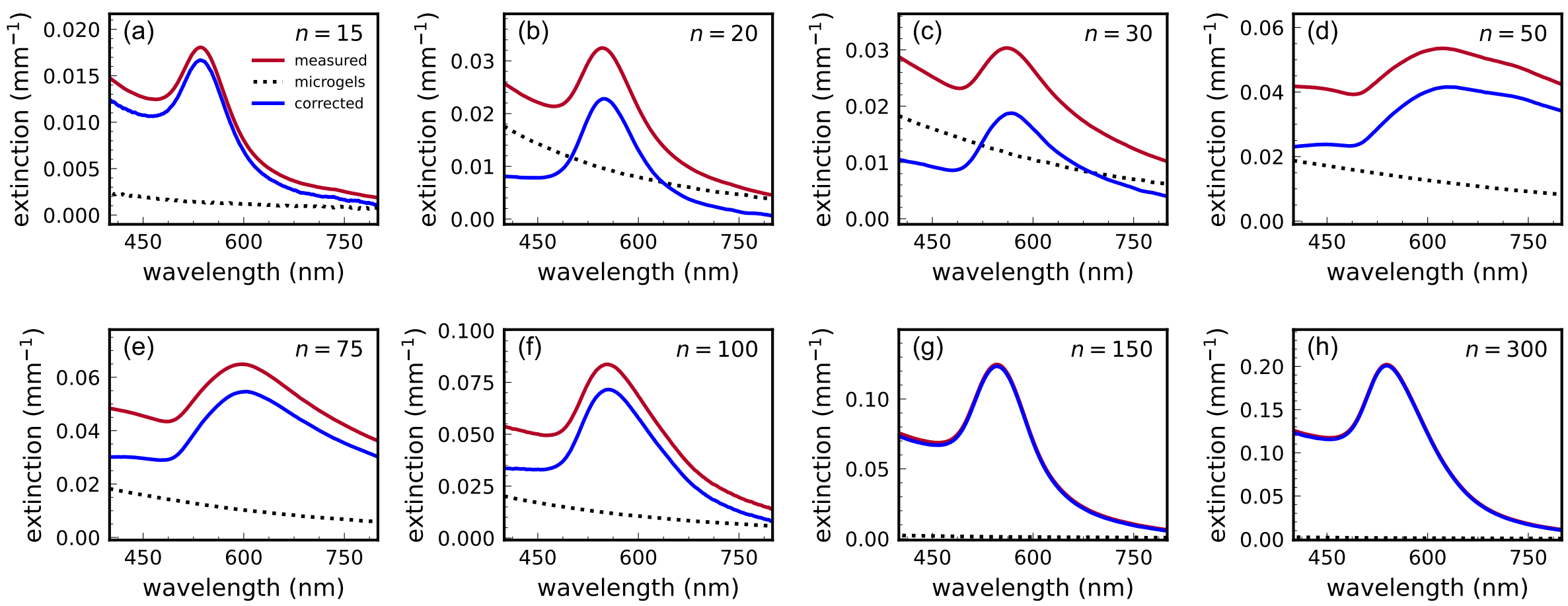

Figure S4 Correction of the extinction spectra for the scattering contribution of NP-free microgel aggregates, for each of the NP-to-microgel number ratios n investigated in this work. In each panel, the red solid line is the measured extinction spectrum of the microgel–NP suspension at 40°C; the black dotted line is the extinction spectrum of a NP-free microgel aggregate sample at 40°C, obtained by salt-induced aggregation and size-matched to the corresponding microgel–NP suspension (same microgel number concentration); the blue solid line is the corrected spectrum, obtained by subtracting the microgel aggregate spectrum from the measured one.

In all cases, the reference aggregate spectra exhibit the expected featureless, monotonically decreasing profile characteristic of dielectric particles. After subtraction, the LSPR peak and the long-wavelength coupled-plasmon shoulder remain clearly resolved, confirming that the spectral features attributed to plasmon coupling in the main text are not an artefact of aggregate scattering. We note that this subtraction procedure is inherently approximate. NP-free microgel aggregates and microgel–NP aggregates with the same R_H may differ in internal structure, compactness, and fractal dimension, and therefore need not have identical scattering cross-sections per unit volume. The corrected spectra should accordingly be regarded as estimates of the plasmonic contribution, rather than exact decompositions.

Fig. S5 compares the n-dependence of $\Delta\lambda_{\mathrm{LSPR}}$ and of $\Delta(A_C/A_{tot})$ before and after the background correction, evaluated at 40°C. In both cases, the corrected values are systematically larger than the uncorrected ones. This outcome is a direct consequence of the monotonically decreasing shape of the aggregate scattering spectrum: removing a background that is relatively larger at shorter wavelengths increases the relative weight of the long-wavelength

shoulder and shifts the apparent peak position to the red. The uncorrected values reported in the main text are therefore conservative underestimates of the true degree of plasmon coupling. Most importantly, the re-entrant dependence of both $\Delta\lambda_{\mathrm{LSPR}}$ and $\Delta(A_C/A_{tot})$ on n – the central result of this work – is fully preserved after the correction, and indeed appears more pronounced.

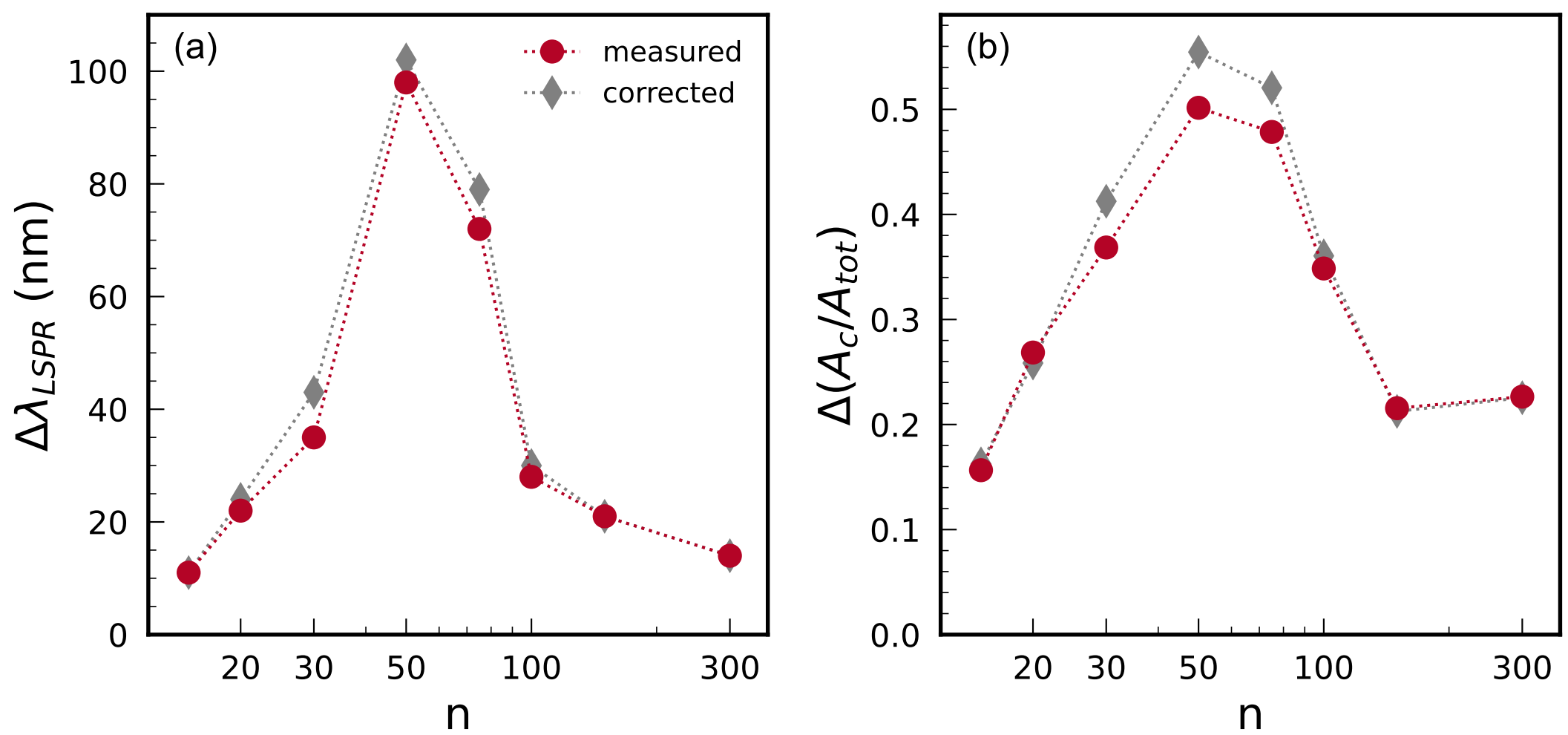

Figure S5 Effect of the aggregate scattering correction on the plasmonic observables at 40°C. (a) LSPR peak redshift $\Delta\lambda_{\mathrm{LSPR}}$ and (b) degree of plasmon coupling $\Delta(A_C/A_{tot})$ as a function of the NP-to-microgel number ratio n, before (red circles) and after (grey diamonds) subtraction of the size-matched NP-free microgel aggregate spectra (see Fig. S4). In both panels, the corrected values are systematically larger than the uncorrected ones. The re-entrant dependence on n, with a maximum at $n = 50$, is fully preserved after the correction.

S4. Assessment of thermal reversibility

Colloidal stability. Fig. S6 shows R_H as a function of temperature during heating (light red triangles) and cooling (light blue triangles). For $n = 15$, 150, and 300, the two branches overlap closely across the entire temperature range, confirming that the VPT of the NP-decorated microgels is thermally reversible and that colloidal stability is maintained throughout the cycle. For intermediate n ($20 \leq n \leq 100$), the cooling branch leads to values of R_H at 25°C far exceeding the initial ones, demonstrating that the charge-patch-driven aggregation is irreversible. We note that this behaviour differs from that observed for microgels complexed with linear polyelectrolyte, where charge-patch-driven clusters can redisperse upon cooling[7]. Whether the irreversibility observed here is due to the higher surface charge density of the NPs, to their larger size, to their different chemical nature, or to a combination of these factors, remains an open question that needs further investigation.

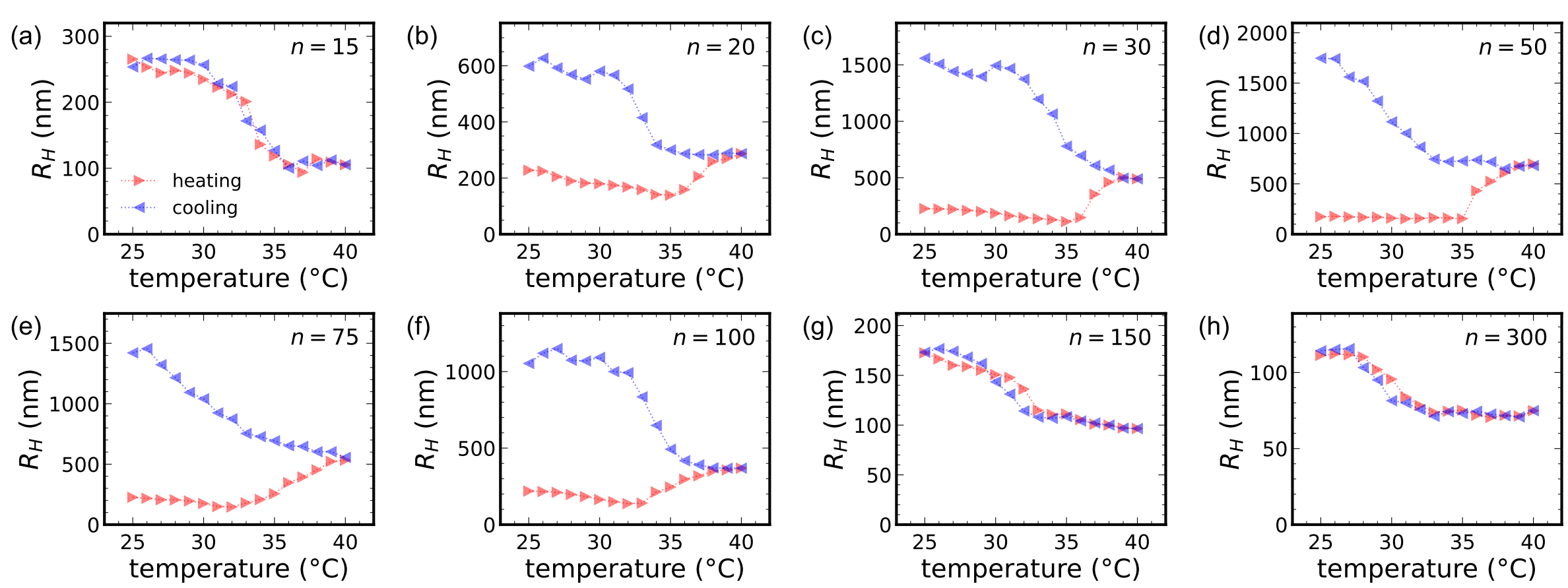

Figure S6 Thermal reversibility of the hydrodynamic radius. R_H is shown as a function of temperature during heating (25°C $\rightarrow$ 40°C, light red triangles) and cooling (40°C $\rightarrow$ 25°C, light blue triangles) for each of the NP-to-microgel number ratios n investigated in this work. For $n = 15$, 150, and 300, the two branches overlap throughout, indicating reversible swelling and deswelling. For intermediate n, the cooling branch reaches values of R_H at 25°C far exceeding the initial ones, demonstrating that the formed aggregates do not redisperse.

Plasmonic observables. Figs. S7 and S8 show $\Delta(A_C/A_{\mathrm{tot}})$ and $\Delta\lambda_{\mathrm{LSPR}}$, respectively, as a function of temperature during heating and cooling. For the aggregating samples ($20 \leq n \leq 100$), both observables display pronounced hysteresis consistent with the irreversible aggregation observed by DLS: the strongly coupled configurations induced by aggregate

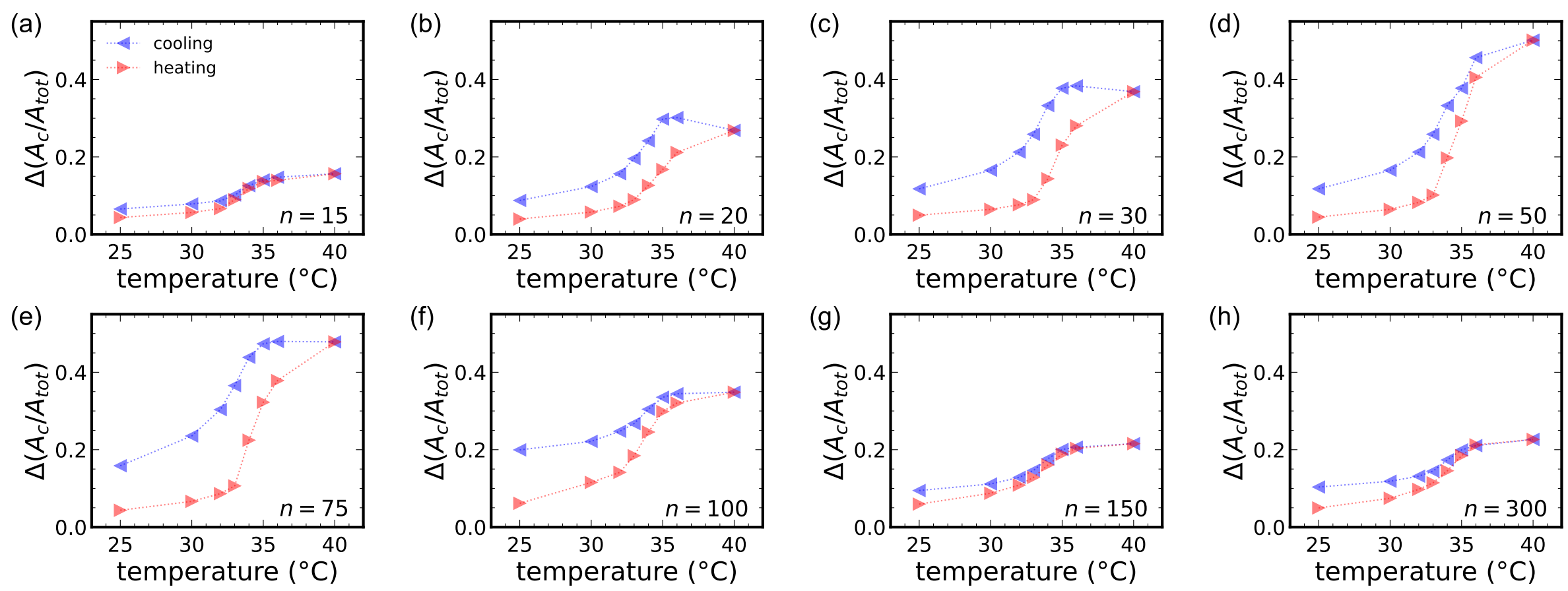

Figure S7 Thermal reversibility of the degree of plasmon coupling $\Delta(A_C/A_{\mathrm{tot}})$. Data are shown during heating (light red triangles) and cooling (light blue triangles) for each of the NP-to-microgel number ratios n investigated in this work. For non-aggregating samples ($n = 15$, 150, and 300), the two branches closely overlap over most of the temperature range, with a small residual offset at 25°C after cooling. For aggregating samples ($20 \leq n \leq 100$), the coupling degree remains elevated upon cooling, reflecting the persistence of strongly coupled NP configurations within the aggregates.

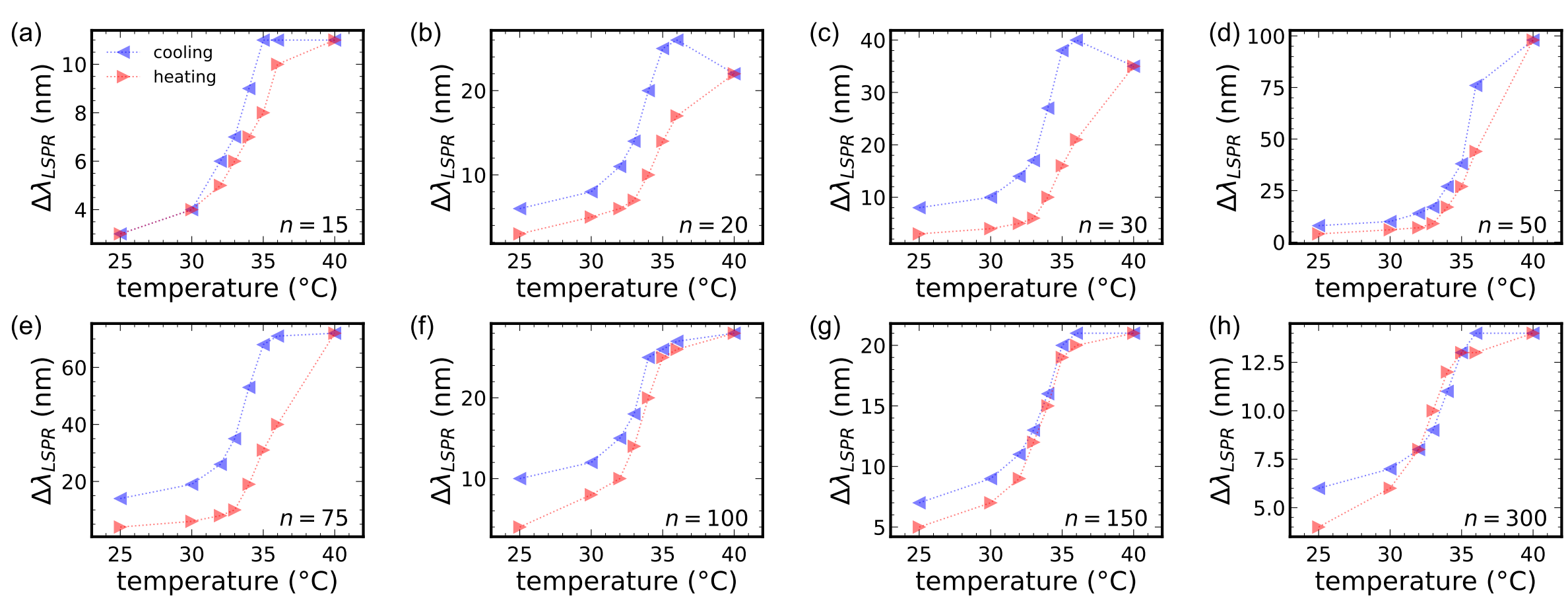

Figure S8 Thermal reversibility of the LSPR peak shift $\Delta\lambda_{\mathrm{LSPR}}$. Data are shown during heating (light red triangles) and cooling (light blue triangles) for each of the NP-to-microgel number ratios n investigated in this work. The behaviour mirrors that of $\Delta(A_C/A_{\mathrm{tot}})$ (Fig. S7): largely reversible for non-aggregating samples with a small residual offset at 25°C, and strongly irreversible for aggregating samples.

formation persist upon cooling. For the non-aggregating samples (n = 15, 150, and 300), the cooling branches closely follow the heating ones over most of the temperature range, but a small residual offset at 25°C indicates that the optical response is not perfectly reversible. To clarify the origin of the residual offset in the plasmonic observables at 25°C for the non-aggregating samples (n = 15, 150, and 300), we compare in Fig. S9 the extinction spectra recorded at 25°C before and after the thermal cycle. We could identify two regimes with different behavior. At $n = 15$ (panel a), the post-cycle spectrum shows a slight reduction of the LSPR peak height with no appreciable increase of the long-wavelength shoulder ($\lambda > 570$ nm). This indicates that the increase in $\Delta(A_C/A_{\mathrm{tot}})$ after the cycle does not arise from near-field coupling between NPs, but rather from a modification of the local dielectric environment of the NPs. We tentatively attribute this to polymer chains from the microgel network that adsorb onto the NP surface during collapse and remain attached after re-swelling, increasing the effective refractive index sensed by the NPs and broadening their LSPR. At $n = 300$ (panel c), and to a lesser extent at $n = 150$ (panel b), the post-cycle spectrum shows instead a clear enhancement of extinction at $\lambda > 570$ nm, with the emergence of a coupled-plasmon shoulder absent before the cycle. This is a direct spectroscopic signature of residual near-field coupling between NPs that have not fully re-separated upon microgel re-swelling. In the high-n regime, the large number of NPs per microgel causes close NP–NP contacts to form during collapse; polymer-mediated interparticle attractions can then stabilize these configurations, preventing complete re-separation upon re-swelling.

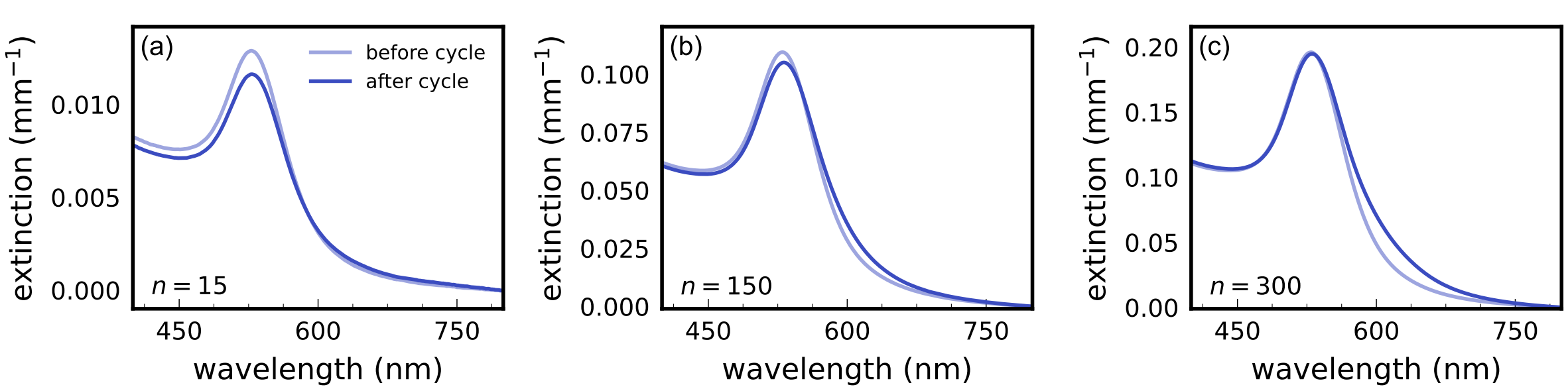

Figure S9 Extinction spectra at 25°C before (light blue) and after (dark blue) a complete heating–cooling cycle for non-aggregating samples: (a) $n = 15$, (b) $n = 150$, and (c) $n = 300$.

References

(1) Otten, M.; Hildebrandt, M.; Pfeffing, B.; Voigt, V. C.; Scheffold, F.; Hellweg, T.; Karg, M. Volume phase transition of thermoresponsive microgels scrutinized by dynamic light scattering and turbidity: Correlations depend on microgel homogeneity. *Langmuir* **2024**, *40*, 14515–14526.

(2) Brasili, F.; Capocefalo, A.; Del Monte, G.; Rivas-Barbosa, R.; Pérez, J.; Chauveau, E.; Bordi, F.; Rizza, C.; Truzzolillo, D.; Zaccarelli, E.; Sennato, S. Linking structure and optical properties of plasmonic nanoparticles on tunable spherical surfaces. *ACS Applied Materials & Interfaces* **2025**, *17*, 47385–47395.

(3) Stout, R. F.; Khair, A. S. A continuum approach to predicting electrophoretic mobility reversals. *Journal of Fluid Mechanics* **2014**, *752*, R1.

(4) Sennato, S.; Chauveau, E.; Casciardi, S.; Bordi, F.; Truzzolillo, D. The Double-faced Electrostatic Behavior of PNIPAm Microgels. *Polymers* **2021**, *13*, 1153.

(5) Brasili, F.; Del Monte, G.; Capocefalo, A.; Chauveau, E.; Buratti, E.; Casciardi, S.; Truzzolillo, D.; Sennato, S.; Zaccarelli, E. Toward a unified description of the electrostatic assembly of microgels and nanoparticles. *ACS Applied Materials & Interfaces* **2023**, *15*, 58770–58783.

(6) Brasili, F.; Capocefalo, A.; Palmieri, D.; Capitani, F.; Chiessi, E.; Paradossi, G.; Bordi, F.; Domenici, F. Assembling Patchy Plasmonic Nanoparticles with Aggregation-dependent Antibacterial Activity. *Journal of Colloid and Interface Science* **2020**, *580*, 419–428.

(7) Truzzolillo, D.; Sennato, S.; Sarti, S.; Casciardi, S.; Bazzoni, C.; Bordi, F. Overcharging and Reentrant Condensation of thermoresponsive Ionic Microgels. *Soft Matter* **2018**, *14*, 4110–4125.
